# The Role of Systemic Physiology in Individual Hemodynamic Responses Measured on the Head Due to Long-Term Stimulation Involving Colored Light Exposure and a Cognitive Task: An SPA-fNIRS Study

**DOI:** 10.3390/brainsci12050597

**Published:** 2022-05-03

**Authors:** Felix Scholkmann, Hamoon Zohdi, Ursula Wolf

**Affiliations:** 1 Institute of Complementary and Integrative Medicine, University of Bern, 3012 Bern, Switzerland; hamoon.zohdi@unibe.ch; 2 Biomedical Optics Research Laboratory, Department of Neonatology, University Hospital Zurich, University of Zurich, 8091 Zurich, Switzerland

**Keywords:** functional near-infrared spectroscopy, fNIRS, systemic physiology augmented functional near-infrared spectroscopy, SPA-fNIRS, colored light exposure, verbal fluency task, multitask paradigm

## Abstract

In our previous investigations using systemic physiology augmented functional near-infrared spectroscopy (SPA-fNIRS) neuroimaging, we found larger variability between subjects in changes of cerebral hemodynamics and oxygenation induced by an intricate experimental paradigm involving colored light exposure and a cognitive task. We aimed to investigate the role the activity of the systemic physiology has on individual variations in the fNIRS data. Thirty-two healthy subjects (17 female, 15 male and age: 25.5 ± 4.3 years) were exposed to blue and red light for 9 min (colored light exposure, CLE) while performing a verbal fluency task (VFT). We found that (i), at the group level, the visual cortex showed a stronger deoxyhemoglobin concentration response during blue light exposure than during red light exposure, and (ii) this relationship was influenced by individually different baseline blood pressure values. Furthermore, we found other correlations between changes in fNIRS signals and changes in systemic physiology. Our study demonstrates the usefulness and necessity of the SPA-fNIRS approach to gain insights into the individual variability of hemodynamic responses measured with fNIRS, especially in the case of an intricate experimental paradigm (i.e., CLE-VFT) as used in our study.

## 1. Introduction

By employing functional near-infrared spectroscopy (fNIRS)-based neuroimaging [1,2,3], our group previously investigated how cerebral hemodynamics and oxygenation change during various paradigms involving short-term or long-term colored light exposures with different colors [4,5,6,7,8,9]. In our latest studies [4,5], we investigated whether a cognitive task (verbal fluency task, VFT) performed under two different color light exposures (CLE; red or blue light) is associated with specific changes in cerebral hemodynamics and oxygenation, measured with fNIRS, as well as specific changes in systemic physiology (e.g., blood pressure or electrodermal activity). We termed this approach “systemic physiology augmented fNIRS” (SPA-fNIRS) [7]. We found large between-subject variability in the changes of the biosignals (fNIRS and systemic physiological parameters) with respect to the two types of light exposures. Large increases in hemodynamics and oxygenation over the prefrontal cortex (PFC) and in heart rate were found in some subjects, while this was not the case in others. We were able to demonstrate by a subgroup analysis that the individual cerebral hemodynamic responses during the CLE-VFT paradigm can be classified into specific reactivity patterns and that the changes in systemic physiology (i.e., arterial oxygen saturation and blood pressure) were related to the magnitude of changes in cerebral hemodynamics and oxygenation (i.e., changes in oxyhemoglobin concentration) in PFC [5]. Supported by another data set of our CLE SPA-fNIRS measurements [6], we were able to validate the observation that individual changes in cerebral hemodynamics and oxygenation during a CLE can be classified into subgroups.

What remains unexplained is why some subjects reacted more strongly to exposure to a specific color (e.g., blue) of light. To answer this question, the aim was to develop and apply a novel data analysis framework to investigate the color-dependent relationships in detail between fNIRS and systemic physiology biosignals in our previous data [4,5] on the subject level.

## 2. Subjects and Methods

### 2.1. Subjects

Thirty-two healthy adults participated in this study (15 men, 17 women, age: 25.5 ± 4.3 years, range: 19 to 45 years). The subjects were asked not to smoke, eat or consume any stimulants such as caffeine or energy drinks for 2 h before the start of measurements. The study was approved by the Ethical Committee of the Canton of Bern, Switzerland. Written informed consent was obtained from the subjects prior to each measurement.

### 2.2. Measurement Setup

SPA-fNIRS measurements were performed by a setup comprising a multi-channel frequency-domain (FD) NIRS device (Imagent, ISS Inc, Champaign, IL, USA) and multiple measurement devices to measure systemic physiological signals. All data were recorded simultaneously.

FD-NIRS enables the measurement of absolute concentrations of oxyhemoglobin ([O_2_Hb]) and deoxyhemoglobin ([HHb]) in the head tissue by optodes that can be placed on the head. Our ISS Imagent device employs 16 laser diodes at 760 nm and 16 at 830 nm and 4 highly sensitive photomultiplier tubes as light detectors. The light sources and detectors are connected with optical fibers to four optodes. Each optode has a specific arrangement of light sources and detectors to enable multi-distance measurements (source-detector separations: 2.0, 2.5, 3.5 and 4.0 cm) [10], which enables more sensitive measurements for brain tissue. The optodes were attached to the head over the left and right PFC (at positions Fp1 and Fp2, according to the international 10–20 system [11]) as well as the left and right visual cortex (VC) (at positions O1 and O2). Interference of the red light exposure with fNIRS measurements was avoided by covering the head of the participant with a thick black blanket.

Heart rate (HR), mean arterial pressure (MAP) and peripheral arterial oxygenation (SpO_2_) were recorded by the SOMNOtouch NIBP (SOMNOmedics GmbH, Randersacker, Germany) device (sampling rate: 4 Hz). Respiration rate (RR) and end-tidal carbon dioxide (P_ET_CO_2_) were measured with the NONIN LifeSense (NONIN Medical, Plymouth, MN, USA) device (sampling rate: 1 Hz). Skin conductance (SC) was recorded with the portable device (Mind-Reflection, Verim, Poland) with two sensors attached to the right index and middle finger.

### 2.3. Experimental Protocol

A detailed description of the experimental protocol can be found in our previous publications [4,5]. Briefly summarized, the protocol consisted of exposing subjects to colored light (illuminance: 120 lux at eye level) for 9 min while having them perform a VFT (9 trials of 30 s duration with 30 s pauses between). The subjects sat upright in a comfortable chair in front of a white wall (distance from the subject to the wall: 160 ± 5 cm) onto which the light was projected. Each subject participated twice (on different days), and they were irradiated with one of the light colors (red or blue, the order of which was randomized). The subjects were instructed to keep their eyes open during the entire experiment. There were rest periods before and after the CLE-VFT at 8 min and 15 min, respectively. During these rest periods, the light was switched off, and the subjects sat in a dark room.

### 2.4. Signal Processing and Statistical Analysis

**Figure 1:**
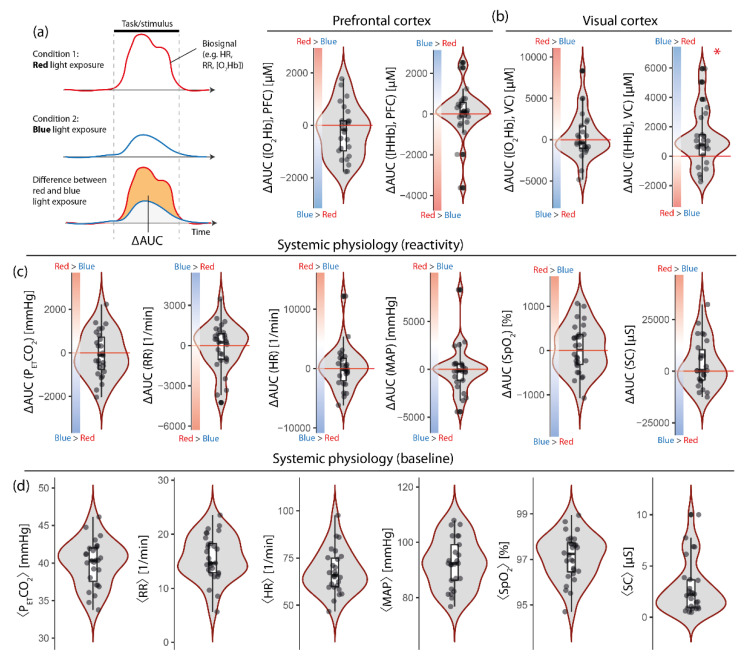
(**a**) Illustration of the calculation of AUC values of the biosignals. (**b**) ∆AUC values for fNIRS biosignals were measured over the prefrontal and the visual cortex. (**c**) ∆AUC values for systemic physiological signals. (**d**) Baseline values (resting-state) for systemic physiological signals. Note: Positive values of ∆AUC correspond to a stronger response of the respective biosignal to the red color exposure compared to the blue one (and vice versa). The ∆AUC and mean values are visualized as box plots with density functions and single data values for each subject added. Asterisk indicates *p* < 0.05 compared to a distribution around zero.

Signal pre-processing was conducted as in our previous study [5]. To investigate if the changes of the biosignals recorded ([O_2_Hb], [HHb], HR, MAP, SpO_2_, RR, P_ET_CO_2_ and SC) were different during the task/stimulation period for the two-color conditions (red vs. blue light exposure), the following approach was used: for each time-dependent biosignal (*x*), the area under the curve (AUC) for the task/stimulation period was calculated for both conditions (AUC (*x*, red), AUC (*x*, blue)), and the difference in AUC was calculated: ΔAUC(*x*) = AUC(*x*, red)–AUC(*x*, blue) (for a visualization see Figure 1a). This calculation was performed for each subject and enabled the investigation of between-subject variability with respect to the color condition. The ∆AUC values, thus, represent how much each individual reacted differentially to red color exposure compared to the blue one.

The ∆AUC from all biosignals was then first analyzed to examine if the distribution of their values for each signal was different from zero (Wilcoxon signed-rank test).

The possible dependence of the fNIRS AUC values ([O_2_Hb] and [HHb] from VC and PFC, respectively) on AUC values from changes in systemic physiology was investigated by linear regression analysis (Pearson correlation). In addition, the mean baseline values of all biosignals were calculated from the baseline period (i.e., from minute 3 to minute 8 of the baseline period before the CLE-VFT task). Furthermore, these baseline values, reflecting the individual resting-state physiology, were analyzed if they were correlated with fNIRS ∆AUC values. 

As in our previous analysis of the data [5], the data from two subjects were removed for correlation analysis in order to obtain a data set for the analysis comprising a homogeneous population with respect to age. The subjects included in the data set were in the age range from 20 to 30 years of age. The exclusion of the two older subjects (aged 32 and 45 years) ensured that age was not a confounding factor in statistical analyses. The data set for the analysis thus comprised 60 data sets (two measurements of 30 subjects).

Signal processing and data analysis were performed in MATLAB (R2017a, MathWorks, Inc., Natick, MA, USA), JASP (jasp-stats.org, version 0.9.2.0) and R (version 4.0.3).

For signal analyses and the description of the results, the recently published fNIRS guidelines [12] were taken into account.

## 3. Results

### 3.1. The Visual Cortex Shows Stronger Response to Blue Than to Red Light Exposure

As Figure 1b shows, the changes in [HHb] at VC were larger during the blue light exposure compared to the red one (*p* = 0.011, effect size (matched rank biserial correlation): 0.569 (95% CI: 0.196, 0.799; Vovk–Sellke maximum *p*-ratio: 7.186)). At VC, [O_2_Hb] did not show a difference between the two light conditions. At PFC, neither [O_2_Hb] nor [HHb] showed color-dependent changes.

### 3.2. Systemic Physiological Reactions Are Not Different for Red Compared to Blue Light Exposure

On the group level, no biosignals from systemic physiology showed a stronger change for one of the two light exposure conditions (Figure 1c), e.g., the red color light exposure did not elicit a stronger increase in blood pressure, MAP, overall.

### 3.3. The Magnitude of Differential Changes in fNIRS Signals (Red vs. Blue Light Exposure) Are Correlated with Baseline Levels and Reactivity of Systemic Physiology

**Figure 2:**
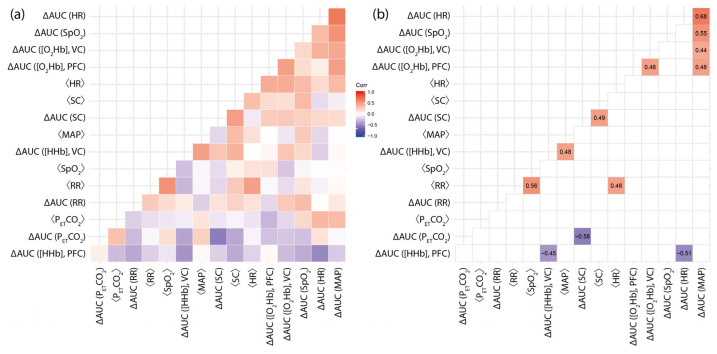
Correlation matrix of the ∆AUC and mean baseline values of all biosignals with (**a**) all values of the correlation coefficient shown and (**b**) with only statistically significant (*p* < 0.05) correlations (the numerical values refer to the correlation coefficient). All statistically significant correlations remained significant after multiple-comparison correction (false discovery rate correction).

**Figure 3:**
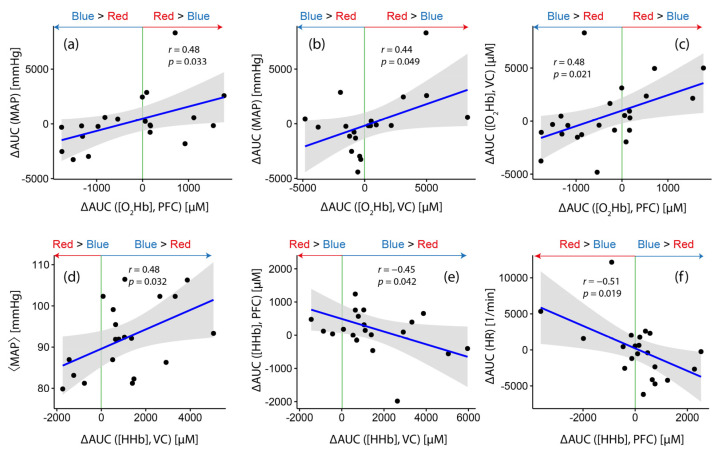
Correlations between ∆AUC values from fNIRS signals and systemic physiological signals. Results of linear correlation analysis. The gray shaded areas refer to the confidence bounds of the determined correlation.

Figure 2 shows six statistically significant correlations between fNIRS parameters (two cases) as well as between fNIRS and systemic physiological parameters (four cases) with respect to the light condition and/or the baseline values. The results can be summarized as follows:

1. While [HHb] at VC, on the group level, showed a stronger increase during blue light exposure (as already discussed in Section 3.1), the magnitude of the differences (∆AUC ([HHb]) was correlated with the baseline MAP level (⟨MAP⟩) (*r* = 0.48, *p* = 0.032; Figure 3d), i.e., the larger the baseline MAP of a subject, the higher the chance to have a stronger increase in [HHb] during blue light exposure compared to red light exposure (and vice versa). Furthermore, ∆AUC ([HHb]) at the VC was negatively correlated with ∆AUC ([HHb]) at PFC (*r* = −0.45, *p* = 0.042; Figure 3e).
2. ∆AUC ([O_2_Hb]) at VC was positively correlated with ∆AUC ([MAP]) (*r* = 0.44, *p* = 0.049; Figure 3b), i.e., subjects with a larger increase in [O_2_Hb] during red light exposure compared to the blue one had a higher probability of also having a larger increase in [MAP] during red light exposure compared to the blue one.
3. For PFC, three statistically significant correlations were found. ∆AUC ([HHb]) was negatively correlated with ∆AUC (HR) (*r* = −0.51, *p* = 0.019; Figure 3f), while ∆AUC ([O_2_Hb] showed positive correlations with ∆AUC (MAP) (*r* = 0.48, *p* = 0.033; Figure 3a) and ∆AUC ([O_2_Hb]) at VC (*r* = 0.48, *p* = 0.021; Figure 3c).

### 3.4. The Magnitude of Differential Changes in Systemic Physiology (Red vs. Blue Light Exposure): Interrelationships and Correlations with Baseline Values

**Figure 4:**
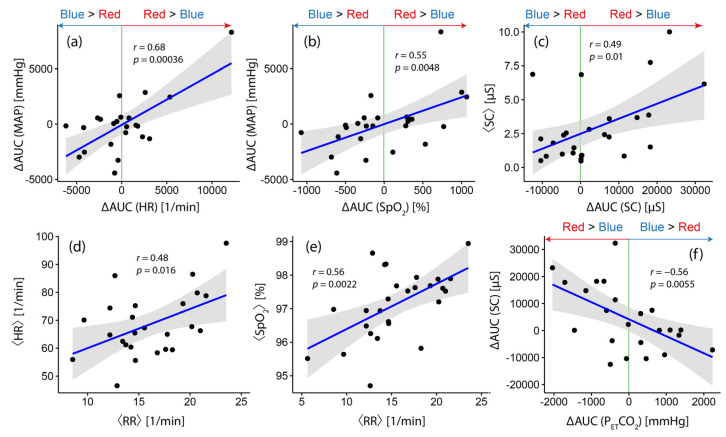
Correlations between ∆AUC values of systemic physiological signals. Results of linear correlation analysis. The gray shaded areas refer to the confidence bounds of the determined correlation.

Concerning color-dependent changes in systemic physiology, six statistically significant correlations were found (Figure 4), which can be summarized as follows:

1. ∆AUC ([MAP]) was positively correlated with (∆AUC ([HR]) (*r* = 0.68, *p* = 0.00036; Figure 4a) and (∆AUC ([SpO_2_]) (*r* = 0.55, *p* = 0.0048; Figure 4b). In the case of a stronger MAP response due to red light exposure compared to blue light exposure, the probability that a stronger response would also be observed in HR and SpO_2_ increased.
2. ∆AUC ([SC]) was negatively correlated with ∆AUC ([P_ET_CO_2_]) (*r* = −0.56, *p* = 0.0055; Figure 4f) and positively correlated with the mean baseline SC level (⟨SC⟩, *p* = 0.49, *p* = 0.01; Figure 4c).
3. The RR baseline level, ⟨RR⟩, was positively correlated with the baseline levels of HR (⟨HR⟩, *r* = 0.48, *p* = 0.016; Figure 4d) and SpO_2_ (⟨SpO_2_⟩, *r* = 0.56, *p* = 0.0022; Figure 4e), respectively. On the group level, subjects with a higher baseline RR also had a higher HR and SpO_2_.

## 4. Discussion

The results of our study provide new insights into how the individual response to a multitask paradigm, involving light exposure with different colors and a VFT, is correlated in a specific manner with individual reactivities and the baseline values of systemic physiology.

### 4.1. The Magnitude of Changes in [HHb] at the Visual Cortex Depends on the Type of Color Exposure and Is Related to the Individual Baseline Blood Pressure Level

According to our study, [HHb] at VC was the most sensitive marker for detecting differences in how cerebral hemodynamics and oxygenation change during the CLE performed with different colors of light while conducting a cognitive task in parallel. A color-dependent change of [HHb] at VC was also found in our previous study [4], which applied a slightly different approach to perform group-based data analyses (i.e., in the previous study, the task period was divided into 3 min long intervals, whereas in the current study, we analyzed the entire 9 min period in total and calculated the AUC). In our previous study, [HHb] was found to also show a stronger decrease at VC during the blue CLE compared to the red one—in agreement with the present finding. Furthermore, the previous analysis did not detect color-dependent changes in systemic physiology, unlike the current study, which found such changes.

Our observation that subjects who showed a stronger change in [HHb] at VC also tended to have a higher baseline MAP level is an intriguing finding. It provides new insights into the inter-subject variability of hemodynamic responses in fNIRS studies, especially when color exposure is involved. The relationship between color-specific changes in [HHb] at the VC and ⟨MAP⟩ might be either an epiphenomenon or a physiologically relevant causal relationship. To answer this question, three additional findings provide new clues: (i) during red light exposure, there was an increase in [HHb] (AUC, median: 510.1, IQR: −372.1, 1087.0), while during blue light exposure, a decrease in [HHb] occurred (AUC, −578.6 (IQR: −868.8, −196.3)); (ii) on the group-level, the ⟨MAP⟩ values for both conditions are not statistically significantly different from each other (blue color exposure: 94.2 ± 9.1 mmHg; red color exposure: 91.2 ± 10.1 mmHg); (iii) on the group level, there is no statistically significant correlation between [HHb] (AUC values) and ⟨MAP⟩: *r* = 0.017, *p* = 0.908. These additional findings support the notion that the relationship between color-specific changes in [HHb] at the VC and ⟨MAP⟩ is not primarily due to a change in blood pressure and, thus, cerebral blood volume affecting the [HHb] signal (confounding effect), but it is rather due to a functional relationship between the baseline blood pressure and neurovascular coupling and/or vascular reactivity (i.e., to a change in systemic physiology) modulated by the task/stimulation. As far as we know, only one functional magnetic resonance imaging (fMRI) study has investigated the effect of baseline blood pressure on task-evoked cerebral hemodynamic responses: Coulson et al. [13] found an inverse relationship between baseline blood pressure and hemodynamic responses in the brainstem in humans during isometric forearm contractions. Interestingly, the significance of the resting blood pressure in functional neuroimaging studies was highlighted in a recent study by Kobuch et al. [14], which reported for the first time that “resting regional cerebral blood flow and functional connectivity levels are significantly related to an individual’s resting mean blood pressure, even in the narrow normotensive range”. Our findings thus provide indications that the measurement of baseline MAP might be relevant for future fNIRS (and fMRI) studies, especially when complex long-term multitask paradigms are employed. It should also be mentioned that the frontal cortex is involved in the function of the autonomic nervous system; for example, the ventromedial PFC is part of the central autonomic network (CAN) [15], and the frontal orbital cortex and the lateral occipital cortex are part of the complex central autonomic network (CCAN) [16]; these are brain regions that we also covered directly or indirectly with our fNIRS measurements in principle.

The absence of significant changes, on the group level, at the PFC might be due to the fact that two physiological processes canceled each other out: an increase in neurovascular coupling due to the VFT and, in parallel, a hypocapnia-mediated decrease in cerebral blood flow and volume due to the speaking involved in the VFT. The strong impact of P_ET_CO_2_ changes on fNIRS signals when speaking has been documented and highlighted already by our group [17]. Even an increased cognitive effort can lead to slight hyperventilation and, thus, corresponding changes in the fNIRS signal [18].

### 4.2. The Magnitude of Color-Dependent Changes in [O_2_Hb] at the Visual and Prefrontal Cortex Is Related to the Individual Magnitude of Color-Dependent Changes in MAP

Although, on the group level, the response magnitude of [O_2_Hb] at the PFC and VC was not dependent on the color of light exposure (*p* > 0.05), the individual response magnitude was dependent on color exposure when considering the individual magnitude of color-dependent changes in MAP. The relationship between [O_2_Hb] and MAP is also evident when analyzing whether an increase in MAP is also associated with an increase in [O_2_Hb]: Our data shows a significant relationship for this association at PFC (*r* = 0.526, *p* < 0.001) and a trend also for VC (*r* = 0.270, *p* = 0.057). These findings indicate that [O_2_Hb] measured at PFC, and to a specific degree also at VC, is influenced by changes in MAP. This is in line with findings that changes in MAP can affect fNIRS signals (confounding effect from systemic physiology) [19]. According to our findings, PFC seems to be more affected by MAP changes than VC.

### 4.3. A Color-Dependent Fronto-Occipital Coupling

Figure 3c,e demonstrate another important finding: The color-dependent changes in [O_2_Hb] at PFC and VC were positively correlated, while those in [HHb] were negatively correlated. Therefore, a stronger hemodynamic change in VC was also associated with a stronger one in the PFC. This observation can be explained by the fact that the brain activity of both areas was stimulated simultaneously by our experimental paradigm and that the respective strength of the stimulation was modulated by light color.

### 4.4. The Magnitude of Color-Dependent Changes in [HHb] at the Prefrontal Cortex Is Related to the Individual Magnitude of Color-Dependent Changes in HR

The inverse relationship between ∆AUC ([HHb]) from the PFC with ∆AUC (HR) (Figure 3f) indicates a relationship between the cardiac system and the color-dependent changes in cerebral hemodynamics and oxygenation. This correlation is also present when analyzing AUC values independently of the type of color exposure—also in this case, a negative correlation was evident (*r* = −0.366, *p* = 0.009) (for [O_2_Hb], this relationship is statistically insignificant, *r* = 0.108, *p* = 0.356). Changes in [HHb] at the PFC thus seem to be color-dependent and modulated by cardiac activity. Functional brain networks CAN [15] and CCAN [16] may also play a role in explaining this finding.

### 4.5. Color-Dependent Changes in Systemic Physiology

The relationships between color-dependent changes in the systemic physiological biosignals shown in Figure 4 demonstrate that, for most parameters, a positive relationship exists, e.g., a stronger response in MAP during red light exposure was associated with a stronger one in HR and SpO_2_. Interestingly, the relationship between ∆AUC ([SC]) and ∆AUC ([P_ET_CO_2_]) was found such that, during the blue light exposure, a negative response in SC was also associated with a negative response in P_ET_CO_2_.

### 4.6. Strengths and Limitations of the Study

The main strength of this study is that our multimodal SPA-fNIRS measurement, combined with an innovative method to analyze the data, enabled the obtainment of novel insights into why people react differently with respect to the color-type exposure of our experimental CLE-VFT paradigm. Furthermore, our fNIRS measurement setup, by employing a multi-channel FD-NIRS system, minimized the impact of scalp blood flow changes on the measured fNIRS biosignals [20,21] and had a reduced sensitivity to movement artifacts compared to single-distance devices [22], which provided an increased chance of detecting a change in the cerebral tissue compartment and not only in the extracellular one. A limitation may be that even more correlations may have been found if the sample size had been larger than in the current study. Our measurement setup only allowed bilaterally recordings at the PFC and VC. Ideally, fNIRS measurements should be made over the entire head in order to better capture hemodynamic and oxygenation changes in the head tissue due to a complex multitask paradigm (triggering brain activity in several regions), as employed in the present study.

One could argue that the individual VFT performance is the main reason for the individual variability of hemodynamic responses measured with fNIRS and in systemic physiology, but our analysis already takes this into account by analyzing the individual correlations between fNIRS signals and systemic signals while removing the variance of individual VFT performances by normalizing the data (i.e., analyzing only the color-dependent changes).

With regard to the reasons for the physiological changes observed during light exposures, light exposure certainly causes changes in cerebral blood flow due to changes in neuronal activity, but other biophysical processes, such as effects due to photobiomodulation [23,24,25,26], are also possible. Further studies are needed to investigate these different causes. However, these different causes are not directly relevant to the new data analysis reported here.

## 5. Conclusions

Our study demonstrates the usefulness and necessity of the SPA-fNIRS approach to understand the measured hemodynamic responses. It provides new insights into the individual variability of these responses in the case of a complex experimental paradigm (i.e., CLE-VFT), as used in our study. One of the main findings of this study is that the hemodynamic response at VC (in [HHb]) relative to CLE-VFT was color-dependent (stronger during the blue light exposure), and this effect was modulated by the baseline MAP value of each subject. This observation can be interpreted as an effect of an interaction of brain physiology with systemic physiology and/or as an influence of systemic physiology on measured fNIRS signals. Both effects are likely to be the case, highlighting the need for the SPA-fNIRS approach to correctly interpret fNIRS signals and to understand effects that would be missed in a more superficial group-level analysis.

Another aspect for explaining individual differences in hemodynamic responses to stimuli or tasks includes individual response patterns that can be detected by performing empirical subgroup analysis, as shown in our recent work [5,6]. How such subgroups, in turn, relate to individual systemic physiology is the subject of our current research.

Our data analysis approach (based on calculating the difference between two conditions) in this study proved useful for further investigating individual color-dependent responses in fNIRS signals and the systemic physiology. This approach can also be used for future fNIRS and SPA-fNIRS studies.

## Data Availability

The data that support the findings of this study are available from the corresponding author upon reasonable request.
